# Imaging of Leukocyte Trafficking in Alzheimer’s Disease

**DOI:** 10.3389/fimmu.2016.00033

**Published:** 2016-02-15

**Authors:** Enrica Pietronigro, Elena Zenaro, Gabriela Constantin

**Affiliations:** ^1^Section of General Pathology, Department of Medicine, University of Verona, Verona, Italy

**Keywords:** Alzheimer’s disease, leukocyte trafficking, two-photon laser scanning microscopy

## Abstract

Alzheimer’s disease (AD) is the most common neurodegenerative disorder and is characterized by a progressive decline of cognitive functions. The neuropathological features of AD include amyloid beta (Aβ) deposition, intracellular neurofibrillary tangles derived from the cytoskeletal hyperphosphorylated tau protein, amyloid angiopathy, the loss of synapses, and neuronal degeneration. In the last decade, inflammation has emerged as a key feature of AD, but most studies have focused on the role of microglia-driven neuroinflammation mechanisms. A dysfunctional blood–brain barrier has also been implicated in the pathogenesis of AD, and several studies have demonstrated that the vascular deposition of Aβ induces the expression of adhesion molecules and alters the expression of tight junction proteins, potentially facilitating the transmigration of circulating leukocytes. Two-photon laser scanning microscopy (TPLSM) has become an indispensable tool to dissect the molecular mechanisms controlling leukocyte trafficking in the central nervous system (CNS). Recent TPLSM studies have shown that vascular deposition of Aβ in the CNS promotes intraluminal neutrophil adhesion and crawling on the brain endothelium and also that neutrophils extravasate in the parenchyma preferentially in areas with Aβ deposits. These studies have also highlighted a role for LFA-1 integrin in neutrophil accumulation in the CNS of AD-like disease models, revealing that LFA-1 inhibition reduces the corresponding cognitive deficit and AD neuropathology. In this article, we consider how current imaging techniques can help to unravel new inflammation mechanisms in the pathogenesis of AD and identify novel therapeutic strategies to treat the disease by interfering with leukocyte trafficking mechanisms.

## Introduction

Alzheimer’s disease (AD) is the most common neurodegenerative cause of dementia in the elderly and is characterized by a progressive deterioration of cognitive functions. The neuropathological features include amyloid beta (Aβ) neuritic plaques, neurofibrillary tangles (NFTs) comprising aggregates of hyperphosphorylated microtubule tau protein, amyloid angiopathy, and the loss of neurons and synapses (1). The major pathogenic concept in the field of AD research is the amyloid cascade hypothesis, which states that the sequence of pathological events leading to AD is characterized by the accumulation of Aβ peptides resulting from the aberrant processing of amyloid precursor protein (APP) and dysfunctional Aβ clearance, followed by the deposition of NFTs and the onset of synaptic dysfunction and neuronal loss. Aβ peptides are formed by the proteolytic cleavage of APP due to the sequential activities of β-site APP-cleaving enzyme 1 (BACE-1) (β-secretase), γ-secretase, and a protein complex with presenilin 1 (PS1) at its catalytic core. Although amyloid plaques and aggregated tau are both part of the neuropathological definition of the disease, numerous studies suggest that soluble oligomeric forms of Aβ and tau are the predominant mediators of cytotoxicity in AD (2).

Alzheimer’s disease pathology is also characterized by an inflammatory response primarily driven by cytokines and the intrinsic myeloid cells in the brain, which are known as microglia (3). It is now widely accepted that microglia-mediated neuroinflammatory responses may promote the neurodegeneration observed in AD (1, 3). Microglial activation precedes neuropil loss in AD patients, and recent genome-wide association studies have revealed that microglial genes, such as *CD33*, *TREM2*, and *HLA-DR*, are associated with susceptibility to the late-onset form of the disease (3). Furthermore, in response to Aβ or NFTs, microglial cells produce proinflammatory cytokines, chemokines, and complement peptides, which can recruit leukocyte subpopulations to the brain. Aβ also stimulates microglia to produce reactive nitrogen intermediates, such as nitric oxide (NO) and reactive oxygen species (ROS), and the resulting oxidative stress induces neuronal damage (3).

Circulating leukocytes may also play a role in the inflammation process during AD. The migration of leukocytes from blood vessels into the central nervous system (CNS) involves a sequence of adhesion and activation events including (1) capture (tethering) and rolling, which are mediated by the interactions between selectins and mucins, and/or between integrins and proteins with immunoglobulin domains; (2) activation induced by chemokines, resulting in the subsequent activation of integrins; (3) arrest mediated by integrins and their counter-ligands; and (4) diapedesis or transmigration (4). Additional steps may include slow rolling, adhesion strengthening and spreading, and intravascular crawling (5). Leukocyte trafficking in the CNS during inflammatory diseases is mediated predominantly by endothelial E-selectin, P-selectin, and their mucin ligands, as well as leukocyte integrins including α4β1 (also known as very late antigen 4, VLA-4) and αLβ2 (also known as leukocyte function-associated antigen 1, LFA-1), which bind the endothelial vascular cell adhesion molecule (VCAM-1) and intercellular cell adhesion molecule (ICAM-1), respectively.

The role of circulating immune system cells in AD-related brain damage is poorly understood, but the use of *in vivo* imaging techniques, such as two-photon laser scanning microscopy (TPLSM), can provide insights into the mechanisms controlling leukocyte trafficking in AD and may lead to the development of novel therapeutic strategies to delay the progression of the disease. In this review, we discuss recent work on the role of circulating leukocytes in AD, highlighting the use of *in vivo* imaging to investigate leukocyte recruitment in the CNS and to study the basis of novel disease mechanisms.

## Leukocyte Trafficking in AD

Monocytes are circulating leukocytes that play an important role in the innate immune response against pathogens. Numerous studies have shown that peripheral myeloid cells can infiltrate brain tissue and reduce the deposition of Aβ plaques (6–9). The entry of monocytes into the CNS is tightly regulated and involves the CC-chemokine ligand 2 (CCL2)–CCR2 axis (10). Aβ is chemotactic for monocytes, and it induces the secretion of proinflammatory cytokines and monocyte transendothelial migration in a blood–brain barrier (BBB) model, in a process that involves the Aβ receptor (RAGE) and platelet endothelial cell adhesion molecule (PECAM-1) expressed on endothelial cells (11, 12). In agreement with these *in vitro* studies, the injection of synthetic Aβ peptides into the hippocampus triggers the trafficking of bone-marrow-derived monocytic cells into the brain, which then differentiate into ramified microglia and penetrate into the core of Aβ plaques (7, 13). Furthermore, recent data indicate that infiltrating monocytes rather than resident microglia express TREM2, a receptor involved in myeloid cell phagocytosis, further supporting the role of peripheral myeloid cells in AD pathogenesis (14). Intravital TPLSM has elegantly confirmed that patrolling monocytes are attracted to and crawl onto the luminal walls of Aβ-positive veins but not Aβ-positive arteries or Aβ-negative blood vessels (15).

Neutrophils are the most abundant population of cells in the blood and are the primary mediators of the innate immune response. Previous reports (16, 17), including our own results (18), have shown that neutrophils do not necessarily need to accumulate in tissues in high numbers in order to induce tissue damage: intravascular adhesion *per se* without transmigration is sufficient to induce endothelial injury. The role of neutrophils in the induction of neuropathological changes and memory deficit in AD models has been demonstrated only recently (19). A higher number of infiltrating neutrophils was observed at the onset of cognitive deficits in 5xFAD and 3xTg-AD mice, especially in the cortex and hippocampus. In corresponding TPLSM studies, neutrophil extravasation was observed at the early stage of the disease inside the cerebral parenchyma, particularly in areas adjacent to vascular Aβ deposits or rich in intraparenchymal Aβ plaques (19). Similarly, Gr1^+^-labeled cells also infiltrate the brain parenchyma of 5xFAD mice and migrate toward Aβ plaques (20). These data, together with previous *in vitro* results, suggest that Aβ plays a role in the chemotaxis and accumulation of neutrophils in the brains of AD mice (21, 22). Furthermore, soluble Aβ oligomers rapidly trigger neutrophil adhesion to integrin ligands *in vitro* and induce the transition of LFA-1 integrin from the low- to the high-affinity binding state, suggesting that Aβ plays a key role in neutrophil intravascular adhesion and migration into the brain during AD (19). Neutrophils migrate into the brains of mice with AD-like disease by using LFA-1 integrin, which controls both intravascular adhesion and intraparenchymal motility. Indeed, the treatment of AD-like transgenic mice with monoclonal antibodies that block LFA-1 integrin or deplete neutrophils at early stages of the disease (when mice start to present memory impairment) mitigated the neuropathological hallmarks of AD and reversed the cognitive deficits. Most importantly from a therapeutic perspective, blocking neutrophil adhesion during the early stages of the disease provided a long-term beneficial effect on cognition in older mice (19).

The presence of neutrophils in the brains of AD subjects was previously suggested by the presence of cells expressing cathepsin G, a protease produced specifically by neutrophils, within the AD brain parenchyma and inside cerebral blood vessels (23). Additionally, the presence of CAP37, an inflammatory mediator expressed in neutrophils, was reported in the blood vessels and hippocampal vasculature of AD patients (24, 25). However, the first definitive evidence for the presence of neutrophils in the AD brain was our confirmation that myeloperoxidase (MPO)^+^ cells are localized in areas with Aβ deposits (19). These cells were typically found around the periphery of Aβ plaques at a distance of <50 μm, and their non-random distribution suggested that Aβ influences the microenvironmental positioning of neutrophils inside the AD brain.

Several reports indicate that T cells also accumulate in the AD brain (26–29). For example, a greater number of activated CD4^+^ and CD8^+^ T cells was observed in the blood, adhering to the vascular endothelium or migrating into the parenchyma of AD patients, compared to healthy controls or patients with other types of dementia (4, 26–29). Notably, the majority of T cells in AD brain tissue are located in the hippocampus and other limbic structures, which are among those regions most affected in AD (28). In support of these data, enhanced activated CD4^+^ and CD8^+^ were recently identified in the cerebrospinal fluid (CSF) of individuals with mild cognitive impairment (MCI) and patients with mild AD, with the proportion of activated CD8^+^ T cells showing the greatest increase (30). In agreement with human data, mouse studies have shown that T cells infiltrate the brains of APP/PS1 mice, and a proportion of these cells secrete interferon (IFN)-γ or interleukin (IL)-17 (31). This suggests that the inflammatory response stimulated by T cells that have migrated into the AD brain may activate microglia and astrocytes and may recruit other inflammatory cells that are potentially harmful to the CNS, thus exacerbating the pathogenesis of AD.

Taken together, the studies discussed in this section suggest that a role for circulating leukocytes in AD is becoming clearer, but further studies are needed to determine the impact of specific immune cell populations on the cognitive deficit and neuropathological changes in AD. In this context, future TPLSM studies may provide key data to increase our understanding of the mechanisms controlling leukocyte trafficking in the AD brain, as well as interactions between migrating leukocytes and CNS-resident cells.

## TPLSM – The General Context

The optical principles of two-photon microscopy are based on the absorption of two longer-wavelength lower-energy photons as a single quantum of energy by a fluorophore, thus promoting an electron to an excited state (32). TPLSM offers several advantages over traditional forms of microscopy for the investigation of living systems because it provides three-dimensional deep-tissue images and single-cell spatiotemporal information that other imaging techniques cannot achieve (33–35). TPLSM is particularly suitable for high-resolution imaging in intact thick tissues, such as whole organs, brain slices, embryos, and live animals (intravital imaging). Extensive tissue penetration is possible due to the reduced scattering of the infrared (IR) excitation light compared to one-photon confocal microscopy. The restriction of two-photon excitation solely to the focal plane provides most of the advantages over traditional confocal microscopy. TPLSM generates fluorescence only within the focal plane, thus substantially reducing photobleaching and photodamage outside the excitation volume (which represents only a small proportion of the overall sample), and thereby prolonging the viability of specimens especially during long-term imaging.

The spatiotemporal dynamics of leukocyte trafficking can be investigated *in vivo* using cutting-edge TPLSM technology (36, 37). This technique has changed our static view of the immune system and allowed the dissection of leukocyte migration behavior and cell–cell contacts, which are fundamental requirements for an effective immune response (38, 39). Several aspects of leukocyte migration that could not be predicted using *in vitro* systems have been identified, thanks to this advanced technology, including leukocyte intravascular crawling and the directional movement and polarization of emigrated leukocytes along an extravascular chemokine gradient (40, 41).

## TPLSM in the AD Brain

Two-photon laser scanning microscopy has recently contributed to several developments in the field of neuroscience, facilitating studies of cell morphology and function in the living brain. TPLSM has become increasingly necessary to study structural and functional changes in the living brain because the imaging of neurons, glia, and vasculature provides new insights into the function of the CNS under physiological and pathophysiological conditions (42–45). Initial studies investigated the structural plasticity of dendritic spines and the axons of pyramidal neurons in the mouse cortex and how their changes could influence long-term information storage (46, 47). TPLSM also revealed the dynamic structure of microglial cells that constantly survey the brain parenchyma and switch their behavior to an activated state immediately after injury (48). In addition to such morphological studies, TPLSM has also been used for calcium imaging, thus improving the analysis of neuronal signaling and plasticity (49, 50).

In the context of AD, intravital imaging in the brain has mainly been used to study amyloid plaque deposition, dendritic spine loss, and microglial aggregation around Aβ plaques (6, 51–55). TPLSM has allowed the repeated visualization of the same amyloid plaques labeled with fluorescent dyes to evaluate their growth in transgenic mouse models of AD (51, 55, 56). A refined TPLSM method was used to define the kinetics of amyloid plaque growth in Tg2576 mice (57). The simultaneous imaging of amyloid plaques and neurons labeled with viral-expressed green fluorescent protein (GFP) in a transgenic mouse model of AD highlighted the detrimental effect of Aβ on the neuronal circuitry (58).

Recent TPLSM studies have confirmed that dendritic spine loss after newborn amyloid deposits persists in the proximity of amyloid plaques in APP/PS1 mice (59). TPLSM imaging has also revealed that the depletion of Aβ in the brains of PDAPP-YFP transgenic mice treated with anti-Aβ antibodies promotes the rapid recovery of existing amyloid-associated neuritic dystrophy *in vivo*, indicating that axonal and dendritic damage is a partially reversible phenomenon (60).

The availability of transgenic mice expressing fluorescent cells, such as microglia, has provided a more intimate view of the interactions between amyloid plaques, neuronal structures, and cells in the brain parenchyma during the progression of AD (61) or astrocytes (62). The rapid appearance of new amyloid plaques induces progressive changes in neurites, manifesting as dendritic and axonal abnormalities, and the activation and recruitment of microglia to areas with amyloid plaques (63).

Two-photon laser scanning microscopy has also facilitated the characterization of impaired microglial functionality near amyloid deposits in AD mice, suggesting a correlation between plaque deposition and microglial behavior (64).

Two-photon laser scanning microscopy analysis of leukocyte trafficking in the inflamed CNS has focused almost exclusively on infections, stroke, and experimental autoimmune encephalomyelitis (EAE) (65–69). However, recent TPLSM studies have shown that circulating leukocytes migrate into the brain parenchyma of AD mice, revealing previously unknown mechanisms of AD pathogenesis and helping to identify new therapeutic strategies for AD (19, 20). Our recent TPLSM experiments have demonstrated that circulating neutrophils arrest and perform intraluminal crawling preferentially inside blood vessels with Aβ deposits, supporting a role for Aβ in leukocyte migration into the brain (19). Furthermore, we have recently shown using TPLSM that neutrophils isolated from LFA-1-deficient mice cannot adhere to the brain vessels and therefore extravasate in the brain parenchyma, suggesting that LFA-1 integrin is a key mediator of neutrophil trafficking in AD and that targeting this adhesion molecule may have a therapeutic effect. Future studies are necessary to better understand the dynamics of immune cell trafficking in AD and TPLSM experiments that allow the imaging and analysis of leukocyte trafficking in the AD brain are discussed below.

## Imaging the Cortex in AD Mice

*In vivo* imaging in the cortex of AD mice can be achieved using deeply anesthetized animals with the head fixed on a stereotaxic device to reduce movement artifacts. Mice in deep anesthesia cannot maintain their core body temperature at 35–38°C. Heat lamps or a heated stage are therefore required to maintain the correct temperature because leukocyte motility is temperature dependent (70). An incision is made along the midline of the scalp to expose the skull, and any fascia overlying the skull is scraped away (71). Two different surgical preparations can be used for intravital TPLSM in the mouse cortex: the thinned skull preparation and open cranial window. Both these methods have advantages and drawbacks depending on the purpose of the investigation.

The thinned skull technique involves thinning the calvarium to approximately 20–30 μm leaving an intact and almost transparent periosteal layer. A circular region of 1–2 mm in diameter is prepared with a high-speed micro drill and/or a stainless steel burr above the somatosensory and motor cortex. Heat and vibration artifacts are minimized during drilling by the frequent application of cold saline solution or artificial CSF. Heating is also avoided by interrupting the drilling every few seconds. Bone dust is removed using compressed air.

**Figure 1:**
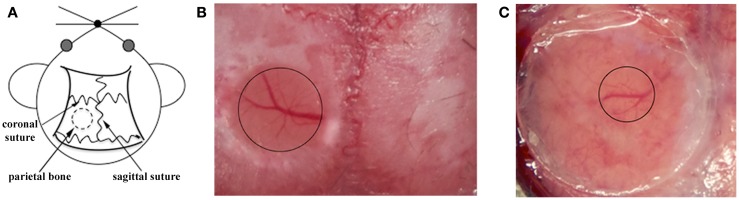
**Surgical procedures for TPLSM imaging of the brain cortex: thinned skull preparation and open cranial window**. **(A)** For both procedures, a circular area of parietal bone is shaved with an electric razor and the scalp is sterilized with alcohol. The head of the anesthetized mouse is then fixed on a stereotaxic device to reduce movement artifacts during imaging, and the skull overlying the cortical region is exposed. **(B)** For the thinned skull preparation, a small region on the parietal zone of the skull is thinned using a high-speed micro drill and a stainless steel burr until the cortical vasculature can be seen clearly through the thinned skull. The thinned area is enclosed by the black circle. **(C)** For the open cranial window technique, a small island of cranial bone on the parietal region (black circle) is drilled, removed with a pair of sharp forceps, and covered with a circular glass coverslip.

The mouse skull consists of two thin layers of compact bone, sandwiching a thick layer of spongy tissue. This spongy bone contains tiny cavities arranged in concentric circles and multiple canaliculi that carry blood vessels. After removing the external compact bone, the middle layer of spongy bone is carefully thinned to approximately 75% of its original thickness. Some bleeding from the blood vessels running through the canaliculi may occur during thinning but usually stops spontaneously (72). The clear visualization of the pial vasculature gives an indication of the skull thickness, then thinning is continued manually using a microsurgical blade. This process is repeated until the bone in the central region becomes flexible and maximum image clarity is achieved (Figures 1A,B). Once the surgical procedure is complete, the thinned skull preparation can be imaged immediately (73). When imaging is completed, the wound margins of the scalp are closed using a nylon suture.

Skull thinning creates a translucent viewing window allowing the visualization of cells up to a depth of 200–300 μm below the pial surface, including the meninges and neocortex but not the deeper brain structures (48). Imaging through the thinned cranial window is a minimally invasive method allowing long acquisition sessions because the thinned skull still protects the brain from external changes in temperature and pressure (74, 75). Despite minor bleeding from diploic vessels, the thinned skull technique leaves the majority of anastomoses between the diploic vessels and dural vessels intact. However, thinning the skull to a specific depth over a large zone is technically challenging due to the curvature of the skull, and the area should therefore be no more than 3 mm in diameter to avoid damage to the underlying tissue (72). Furthermore, in the case of multiple acquisition sessions over time, re-thinning the excess bone deposition after few days is necessary to prevent skull regrowth occluding the preparation (73). Among the two available techniques, the thinned cranial preparation is better for the analysis of larger structures, such as cerebral vasculature, amyloid plaques, and leukocytes (19, 51, 52, 76).

The study of AD mechanisms in mice may benefit from the creation of a small break with the tip of a needle in the lateral wall at the site of the thinned skull preparation to allow the delivery of fluorophores into the brain, leaving the thinned region intact. The hole is then filled with sterile bone wax, and the animals are allowed to recover on a heating pad before being returned to their cages. This approach can be used, e.g., to allow the diffusion of anti-Aβ antibodies directly labeled with fluorescein or thioflavin S, a sensitive and specific fluorescent reporter for the dense-core subset of senile plaques. This molecule has been used to label Aβ deposits in transgenic mouse models of amyloid deposition, and the growth rate of Aβ plaques and cerebral Aβ angiopathy have been extensively monitored *in vivo* (51, 55, 76–78).

The *in vivo* imaging of senile plaques in AD mice can also be achieved using Methoxy-X04, which can be administered intravenously or intraperitoneally (79). Methoxy-X04 is a relatively small, lipophilic molecule that can enter the brain rapidly and in sufficient amounts to allow the sensitive and specific detection of Aβ deposits (79). Senile plaques and cerebrovascular Aβ angiopathy in AD-like mice are visible 30 min after intravenous injection or 24 h after intraperitoneal injection (79). The intravenous or intraperitoneal administration of Methoxy-X04 is a more physiological approach to label Aβ *in vivo* compared to the creation of a small break in the skull near the thinning region to allow delivery of fluorophores into the brain.

The second major approach for TSPLM imaging is the open cranial window. The most challenging aspect of this technique is the surgical skill required for a successful preparation. In this procedure, a circular groove is drilled on the parietal region of the skull, and the island of cranial bone is carefully removed with a pair of sharp forceps. These should be held parallel to the skull surface as far as possible to avoid mechanical injury, because the dura can be attached to the overlying bone. Immediately after removing the region of the skull, slight bleeding above the dura may occur from small blood vessels attached to the removed skull fragment, but this should stop spontaneously within few seconds. The exposed brain tissue is preserved using a drop of 0.9% NaCl. A circular glass coverslip (5 mm in diameter) is placed over the incision site to cover the window (Figure 1C). After cementing the coverslip in place, mice are given a bolus of warm saline for rehydration and are allowed to recover from anesthesia for 7–14 days before imaging. The open cranial window technique also makes it unnecessary to introduce a needle-hole in the skull to deliver stains and labeling compounds. A recently described simple device provides easy physical access to the brain and allows long-term imaging (80). After the initial open cranial window surgery, a glass coverslip is applied as described above, but this has a pre-drilled access hole sealed with biocompatible silicone. This device allows many different types of manipulation to be carried out for weeks or months, e.g., drug, dye, and virus delivery, sample extraction, or electrophysiological recording and stimulation, while protecting the brain from infection (80).

The open cranial window provides improved optical access to the cortical layers, allowing repeated high-resolution imaging, and is preferable for the imaging of small structures, such as dendritic spines and filopodia (73, 81). However, removing the skull may induce a neuroinflammatory reaction and unavoidable meningeal vascular injury, involving the activation of microglia and astrocytes in the intact brain (72, 73, 81, 82). In this case, only cells lying deeper than 80 μm below the pial surface should be considered for image analysis to eliminate possible artifacts caused by the surgical preparation.

To conduct multiple imaging sessions, some authors place a small metal bar containing a hole for a screw next to the coverslip to allow for repositioning of the mouse during subsequent imaging sessions. This approach has been used in Tg2576 mice for the long-term *in vivo* imaging to monitor individual amyloid plaques stained with Methoxy-X04 over a period of 6 weeks (55). Other studies using the same technique have followed individual Aβ plaques in Tg2576 mice for 5 months, confirming the biophysical model of Aβ plaque growth *in vivo*, which had been extrapolated from *in vitro* experiments.

**Figure 2:**
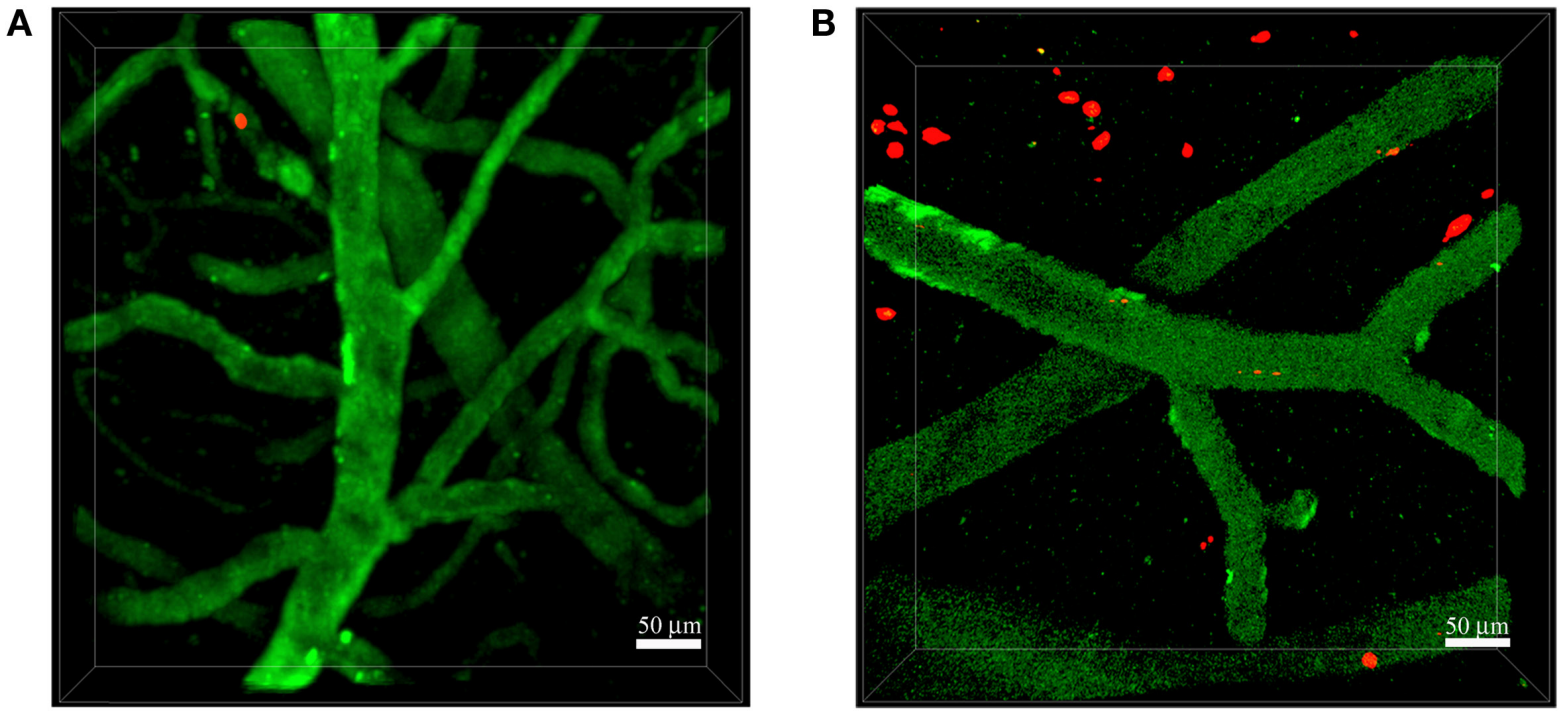
**Neutrophils invade the brain of 5xFAD mice**. Representative TPLSM images of wild-type control mice **(A)** and 5xFAD mice **(B)** showing blood cortical vessels labeled in green using 525-nm non-targeted Qdots injected before image acquisition and neutrophils labeled in red with the fluorescent cell tracker CMTPX. The skull was exposed above the somatosensory cortex using the thinned skull preparation. We performed acquisition inside the brain parenchyma at a depth of approximately 150–250 μm. Images were acquired 16–48 h after cell injection. **(A)** Neutrophils did not interact with the endothelium of blood vessels in wild-type control mice. **(B)** Numerous neutrophils migrated into the brain parenchyma of 5xFAD mice. Scale bars = 50 μm.

Blood vessels can be labeled by the intravenous injection of fluorescent high-molecular-weight dextran (>2000 kDa) or semiconductor nanocrystals (quantum dots), which suffer less interstitial leakage than dextrans (83) (Figure 2). Leukocytes can be labeled *ex vivo* with vital dyes, such as 7-amino-4-chloromethylcoumarin (CMAC, blue), 5- (and 6-) carboxyfluorescein-diacetate succinimidyl-ester (CFSE, green), 5- (and 6-) (((4-chloromethyl)benzoyl)amino)tetramethylrhodamine (CMTMR, orange), C_42_H_40_ClN_3_O_4_ (CMTPX, red), and C_33_H_24_N_2_O_9_ (SNARF-1, far red). The cells can then be adoptively transferred to syngeneic recipients allowing tracking for short time periods, usually a few days (Figure 2). Alternatively, the use of donor mice expressing fluorescent proteins ubiquitously is an option for the *in vivo* analysis of highly proliferative cell populations (84). Similarly, mice expressing fluorescent cell subsets allow the study of specific cell populations, such as neutrophils, lymphocytes, microglia, or macrophages (61, 85–87). Nowadays, mice engineered for the lineage-specific expression of GFP derivatives, such as eCFP and eYFP, or dsRed derivatives, such as tdTomato and mCherry, are widely used because they are less susceptible to phototoxicity and there is no need for cell isolation and labeling. However, most of these mice have been generated using GFP derivatives, limiting the ability to analyze several reporter genes in the same mouse simultaneously. Hence, new IR fluorescent dyes have been developed, which also facilitate deeper tissue imaging (88). The IR excitation wavelength requires the application of an optical parametric oscillator (OPO) to TPLSM. This allows live imaging for an extended duration because it reduces the photobleaching of fluorophores and phototoxicity-induced tissue damage.

**Figure 3:**
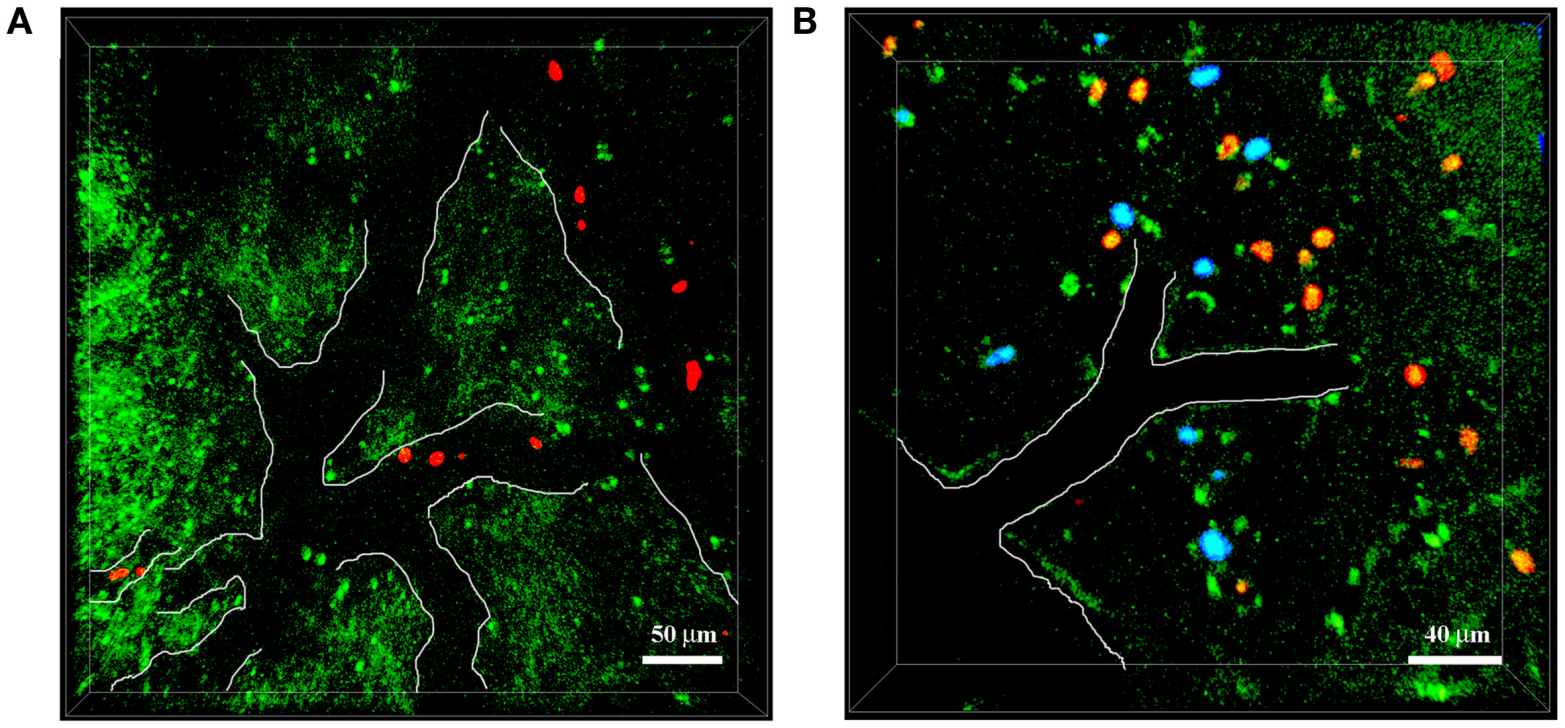
**Neutrophils infiltrate the brain of 5xFAD–YFPH mice in Aβ-rich areas**. Representative TPLSM images of cortical regions in 5xFAD–YFPH mice showing YFP neurons in green, neutrophils in red stained with cell tracker CMTPX, and Aβ plaques in blue labeled by the intravenous injection of Methoxy-X04. The vessel edges are traced artificially with a white line to show the vessel bed. **(A)** Neutrophils interact with the vascular endothelium. Scale bar = 50 μm. **(B)** Neutrophils infiltrate into the brain parenchyma, characterized by abundant Aβ plaques and weak neuronal fluorescence. Scale bar = 40 μm.

The simultaneous expression of fluorescent proteins or staining using fluorescent dyes with different excitation spectra in the same mouse allows the *in vivo* visualization of interactions between specific cell populations and Aβ deposits, thus enabling the study of AD-specific cellular dynamics in the brain. This approach was recently used by our group to demonstrate that neutrophils migrate inside the parenchyma of 5xFAD–YFPH mice in areas with Aβ plaques labeled with Methoxy-X04, probably driven by chemotactic factors, potentially including Aβ peptides (19) (Figure 3). Our TPLSM studies also showed that the extravasation of neutrophils inside the brain of 5xFAD–YFPH mice occurs in areas depleted for eYFP fluorescent neurons (Figure 3). We speculate that eYFP fluorescent neurons are turned off in the zones with neutrophil infiltration, probably due to the cytotoxic effects of proinflammatory mediators released by neutrophils, such as ROS, enzymes, neutrophil extracellular traps (NETs), and cytokines. Alternatively, this may reflect an indirect negative effect caused by the activation of microglia. Moreover, we have shown that neutrophils display arrest and intraluminal crawling preferentially inside blood vessels with labeled Aβ deposits and that some cells undergo diapedesis adjacent to vascular Aβ deposits (19). TPLSM experiments performed by others have shown that neutrophils are attracted inside the brain parenchyma by chronic Aβ deposition – initially the cells move randomly outside the vessels and then they are suddenly and massively recruited to specific Aβ plaques in the brain parenchyma (20).

Two-photon laser scanning microscopy can be used to study CNS-resident cells (such as neuronal subsets, astrocytes, microglia, and perivascular macrophages) in relation to each other or in relation to infiltrating leukocytes. TPLSM studies have shown that local resident microglia react to Aβ plaque formation by extending processes and subsequently migrating toward plaques in APP/PS1 mice crossed with mice containing fluorescent microglia (53). Furthermore, astrocytes in the mouse neocortex can be visualized *in vivo* by intravenous injection of the non-toxic molecule sulforhodamine B or by using transgenic mice, in which astrocytes express enhanced GFP under the control of the mouse glial fibrillary acidic protein (GFAP) promoter (89, 90). TPLSM *in vivo* imaging of astrocyte Ca^2+^ signaling revealed abnormalities in astrocyte activity that may contribute to vascular instability and thereby to neuronal cell death in several transgenic mouse models of AD (91). However, whether infiltrating leukocytes interact with resident neural cells is completely unknown in AD and future studies focusing on such interactions will be necessary to discover and understand these new disease mechanisms.

## Image Acquisition and Analysis

During image acquisition, mice are deeply anesthetized with isoflurane and the rate of respiration is controlled. The laser intensity and photomultiplier tube (PMT) gain need to be set carefully to minimize photodamage. The imaging volume and sampling frequency must be chosen to ensure successful image analysis with fine resolution in time and space. Stacks of optical sections are serially re-acquired at defined time intervals, and cell centroids need to be determined in order to track cell motion. The tracks locate the cells at each time point. They consist of serial sets of *xyz* coordinates of single-cell centroids. The tracks are then exported as numerical data and are used to calculate specific parameters for the analysis of cell migration (36). Specialized software packages are used for automated 3D cell tracking and the analysis of migration paths for each cell. The automatic tracking and analysis of individual cells is more reliable when a small proportion of cells is labeled, because this reduces the likelihood of overlapping pixels representing different cells and allows the distinction of individual cells based on their centers of mass.

Several parameters can be used to analyze the migration behavior of cells during TPLSM experiments. Cell velocity can be represented as either instantaneous velocity or track velocity. Instantaneous velocity is a basic parameter derived from the displacement of the cell divided by the elapsed time (70). The track velocity is calculated from the median or mean instantaneous velocity computed from all time intervals throughout a track, typically 6–14 time points at intervals of 20–50 s (92). Because leukocytes migrate along stromal cell networks, they do not travel along a linear path but instead make frequent turns in a “random walk.” Therefore, higher sampling frequencies are necessary to provide an accurate readout of instantaneous velocities and to avoid the underestimation of real velocity when cells are persistent for only a finite period of time (93). It is easy to find differences in the motility parameters of leukocytes reported in previous studies, and such differences are likely to reflect the frame rate of data acquisition. If there is a long gap between each frame, then the true velocity of the cell will be underestimated. Therefore, the “fast sampling” theory (the use of sampling intervals shorter than the persistence time of the cell) should be taken into account in order to correctly compare data obtained in different laboratories (93).

A useful parameter that is derived from velocity is the arrest coefficient, representing the fraction of time during tracking in which a cell does not move (threshold < 2 μm/min). It is calculated as the ratio of the time a cell is immotile over the whole observation time. The arrest coefficient is calculated from cell tracks and reported as the percentage of cells in the entire population (70). The arrest coefficient is high when leukocytes are in stable contact with other cells or when they swarm in a chemoattractant microenvironment (94).

The cell locomotion parameter allows some speculation on the nature of cell movement, e.g., cells may show directed migration along a gradient of a soluble or surface-immobilized chemoattractant. The displacement of a cell moving with a constant velocity is the shortest distance between the positions at two time points (95). An accurate parameter to analyze migration patterns is the mean displacement (MD) plot (70), in which the average displacement of a population of cells over specific time intervals is plotted against the square root of time. The slope of the resulting curve can be used to determine the motility coefficient of a cell population and measures the volume that a cell scans per unit time. Cells usually exhibit directed movement for a few minutes, which means that during short time intervals, they tend to move in one preferred direction (36).

The chemotactic index, also called the meandering index or straightness index, is another parameter representing the confinement of cell tracks and is calculated based on the ratio of displacement from origin by track length. The meandering can vary between 0 (the cell returns to its exact starting position) and 1 (a perfectly straight cell track). Cells exhibiting frequent angle changes will produce tracks with low meandering indices, whereas a meandering index of 0.7–1 generally shows that cell migration has a strong directional bias (70).

During time-lapse acquisition, a moving cell is observed by taking snapshots at fixed time intervals; hence, the movement of each cell might be considered as a sequence of vectors. Trajectory vectors represent the direction of displacement of individual cells, so the calculation of angles between the direction of migration and various other directions in space is possible, e.g., the orientation of a blood vessel. The vector-vessel angles of individual tracks are useful for the description of tangential movement along an axis and are determined by mirroring the trajectory vectors onto the nearest vessel (reference vector), resulting in angles of 0–90° for each cell track (96).

It is important to realize that cell populations analyzed using the parameters described above may consist of distinct subpopulations that have different biological roles and migration behaviors. These subpopulations may be revealed by plotting the distribution of the parameter of interest among all cells or by studying correlations between multiple motility parameters. For example, neutrophils invading the brain parenchyma in AD-like mice show two distinct behaviors: the first involves a strong directional bias characterized by a high meandering index, motility coefficient, and mean track velocity, whereas the second is characterized by low motility and undirected movement resembling swarming behavior (19). The undirected motility behavior with numerous cells displaying full arrest indicates the presence of activating stop signals for neutrophils in the brains of mice with AD-like pathology, whereas the directed movement of neutrophils strongly suggests the presence of chemotactic factors (19).

Other parameters calculated from leukocyte intratissue migration may reveal whether a chemoattractant released by injured tissue, resident cells, or recruited blood-derived leukocytes direct their movement. For example, after entering peripheral tissues, neutrophils assume an amoeboid motility profile characterized by coordinated migration along a chemokine gradient and cluster formation. The dramatic changes in neutrophil morphology can be analyzed by shape index (length/width), revealing the significant elongation of cell bodies, which is the characteristic of adhesion-independent movement associated with a low degree of cytoskeletal organization and a lack of discrete focal contacts (97).

The directional component of migration can be determined by measuring the turning angles of neutrophil tracks, which is achieved by calculating the angle change between vectors constructed from each time point. The peak angle distribution in absolute values varies in the range 0–180°, and changes of 10–30 units indicate highly directed migration following a chemotactic gradient (97). In order to identify specific chemotactic molecules that direct leukocyte movement, instantaneous radial velocities can be calculated for the comparative chemotaxis analysis of wild-type and gene-deficient neutrophil populations (98). The radial velocity–time plot with fitted regression lines can provide insight into the recruitment dynamics of cell populations, and a reduction in radial velocity may be a consequence of impaired chemotaxis (98). Although no data are yet available in AD models, integrin-dependent intravascular neutrophil migration in sterile liver injuries requires CXCR2 ligands on liver sinusoids and formyl peptides in the injury zone (99). Furthermore, in sterile skin injuries, leukotriene B4 acts as an intercellular signal between neutrophils that allows rapid integrin-independent neutrophil recruitment throughout the tissue (98). Future studies are needed to characterize the dynamics of neutrophils as well as other leukocyte populations in the brains of AD models.

## Future Directions

Two-photon laser scanning microscopy has made it possible to study leukocyte recruitment in the living brain, improving our understanding of immune cell functions in CNS diseases, such as AD. Advanced imaging techniques will also allow us to define the role of leukocyte subpopulations at different stages of disease, as well as the relationships among migrating leukocytes, amyloid deposition, and tau pathology. Moreover, it is conceivable that TPLSM in the AD brain will help us to visualize and analyze the interplay between migrating leukocytes and resident cells, such as microglia, neurons, and astrocytes. TPLSM could also be used to unravel the role of toxic molecules, such as ROS and NO intermediates that may be produced by invading innate immunity cells during AD. For example, dihydroethidium and hydroethidine are cell-permeable fluorescent dyes suitable for the evaluation of ROS synthesis *in vitro*, but they have also been used successfully to visualize anion superoxide production *in vivo* (100, 101). The development of new fluorescent probes and the deep-tissue imaging capability of TPLSM may allow the *in vivo* visualization of ROS production by neural cells and infiltrating leukocytes.

One of the limitations of current imaging studies is the lack of access to deeper areas of the intact brain, such as the hippocampus, which is strongly affected during AD. Nevertheless, recently developed micro-optics and micro-mechanical components have improved deep-tissue imaging (102, 103). Indeed, the use of micro-prisms and gradient index (GRIN) lenses allows the long-term imaging of deep-layer cortical tissue in the mouse brain, although such devices must be inserted into the parenchyma, potentially altering neural cell functions and inducing inflammatory responses (102, 104). GRIN lenses feature plane optical surfaces and are focused by continuously changing the refractive index within the lens material by eliminating aberrations typically found in traditional spherical lenses. An alternative deep-tissue imaging method is stimulated emission depletion (STED) laser microscopy, which allows the exploration of deep areas, such as the hippocampus in living brains (105). This recent imaging technique improves spatial resolution by quenching the fluorescence everywhere except the central region, thus substantially reducing the size of the original fluorescence spot. Therefore, the higher peak signal-to-background ratio reveals more detailed structures, improving the quality of the images. In the near future, STED laser microscopy may expand to include the analysis of leukocyte recruitment in key brain areas during AD, leading to the identification of new disease mechanisms. Moreover, STED technology brings *in vivo* super-resolution microscopy to small structures involved in the morphofunctional interactions between astrocytes and neurons, suggesting future exciting opportunities for the study of leukocyte interactions with neural cells (106).

Overall, the augmentation of TPLSM using optical systems, such as GRIN lenses or STED laser microscopy, and advances in fluorescent dye and protein engineering to produce brighter and more photostable fluorophores will play an important role in the study of molecular mechanisms controlling leukocyte-dependent brain injury and will help to identify new therapeutic approaches for AD.

## Ethics Statement

All mouse experiments were carried out in accordance with guidelines prescribed by the Ethics Committee for the usage of laboratory animals for research purposes at the University of Verona and by the Italian Ministry of Health.

## Author Contributions

All authors contributed equally.

## Conflict of Interest Statement

The authors declare that the research was conducted in the absence of any commercial or financial relationships that could be construed as a potential conflict of interest.
